# Comparison of the Transcriptomes of Mouse Skin Derived Precursors (SKPs) and SKP-Derived Fibroblasts (SFBs) by RNA-Seq

**DOI:** 10.1371/journal.pone.0117739

**Published:** 2015-02-26

**Authors:** Yujie Mao, Lidan Xiong, Siyu Wang, Jianqiao Zhong, Rongying Zhou, Li Li

**Affiliations:** 1 Department of Dermatology, West China Hospital, Sichuan University, Chengdu, 610041, China; 2 Department of Dermatology, Sichuan Academy of Medical Sciences and Sichuan Provincial People’s Hospital, Chengdu, 610041, China; 3 Department of Dermatology, Affiliated Hospital of Luzhou Medical College, Luzhou, 646000, China; New Jersey Institute of Technology, UNITED STATES

## Abstract

Skin-derived precursors (SKPs) from dermis possess the capacities of self-renewal and multipotency. *In vitro* and *in vivo* studies demonstrated that they can differentiate into fibroblasts. However, little is known about the molecular mechanism of the differentiation of SKPs into fibroblasts. Here we compare the transcriptomes of mouse SKPs and SKP-derived fibroblasts (SFBs) by RNA-Seq analysis, trying to find differences in gene expression between the two kinds of cells and then elucidate the candidate genes that may play important roles in the differentiation of SKPs into fibroblasts. A total of 1971 differentially expressed genes (DEGs) were identified by RNA-Seq, which provided abundant data for further analysis. Gene Ontology enrichment analysis revealed that genes related to cell differentiation, cell proliferation, protein binding, transporter activity and membrane were significantly enriched. The most significantly up-regulated genes *Wnt4*, *Wisp2* and *Tsp-1* and down-regulated genes *Slitrk1*, *Klk6*, *Agtr2*, *Ivl*, *Msx1*, *IL15*, *Atp6v0d2*, *Kcne1l* and *Thbs4* may play important roles in the differentiation of SKPs into fibroblasts. KEGG analysis showed that DEGs were significantly enriched in the TGF-β signaling pathway, Wnt signaling pathway and Notch signaling pathway, which have been previously proven to regulate the differentiation and self-renewal of various stem cells. These identified DEGs and pathways could facilitate further investigations of the detailed molecular mechanisms, making it possible to take advantage of the potential therapeutic applications of SKPs in skin regeneration in the future.

## Introduction

Recent developments in stem cell biology have generated much excitement about the potential for regenerative medicine and cell-based therapies in a variety of clinical applications, such as treating leukemia, Parkinson’s disease and wounds. As skin is easily accessible for autologous transplantation, stem cells isolated from skin could be promising candidates for prospective therapeutic applications.

Skin-derived precursors(SKPs) from dermis possess the capacities of self-renewal and multipotency [1–2]. They can differentiate into cells of both neural and mesodermal lineages, such as neurons, glias, smooth muscle cells, osteogenic and adipogenic cells [1–5]. It has also been reported that SKPs can differentiate into fibroblasts. When attached onto culture dishes by serum, SKPs initiated differentiation and developed into a fibroblast-like morphology. These SKP-derived fibroblasts(SFBs) could express fibroblast markers fibronectin and vimentin, but did not express SKP marker nestin [6]. *In vivo* experiments demonstrated that SKPs which were transplanted into dermis became morphologically similar to the endogenous fibroblasts at 2–3 weeks post-transplant and expressed dermal fibroblast markers, but did not express markers of neurons or peripheral glia [7–8]. As the predominant cells in dermis, fibroblasts play a pivotal role in maintaining the form and function of skin. The loss or function impairment of fibroblasts is mainly caused by aging or by injury. Given that SKPs can differentiate into fibroblasts, they might be useful for treating aged skin or regenerating skin after damage since they can replenish lost or damaged fibroblasts.

A complex interplay between the intrinsic genetic processes of stem cells and their environment, including the effects of specific cytokines, determines whether they self-renew, remain quiescent, proliferate, differentiate, or undergo apoptosis. Hence, understanding the nature of SKPs and the molecular process by which these cells differentiate into fibroblasts is crucial for the success of cell based therapies. As the phenotype of any given cell is ultimately the product of its genes, it is necessary to identify gene expression of the cells we are interested in, which for the present study are SKPs and SFBs.

RNA-seq is a high-throughput sequencing platform that can be used for discovery and quantification of transcripts in a single experiment [9–12] and reveal differential expression between different samples [13]. Recent studies have shown RNA-seq to be more accurate over a larger dynamic range of gene expression than microarrays [14–15].

No studies performed with mice employing a transcriptome comparison between SKPs and SFBs have been reported. Therefore, the aim of this study is to compare the transcriptomes of mouse SKPs and SFBs by RNA-Seq analysis. Such an analysis would help determine the candidate genes that might play important roles in the differentiation of SKPs into fibroblasts, explain the molecular mechanisms, and then accelerate the therapeutic application of SKPs in skin.

## Materials and Methods

### Cell Isolation

BALB/c mice at postnatal day 3 for cell isolation were purchased from the Center of Experimental Animal, West China Hospital, Sichuan University. All animal procedures were approved by the Institutional Animal Care and Use Committee of Sichuan University(2013006A). The protocol for isolating SKPs has been described previously in detail [16]. Briefly, dorsal skin was dissected from neonatal BALB/c mice and cut into 2–3 mm^2^ pieces. These dissected pieces were washed three times with Hank’s balanced salt solution(Invitrogen, USA) and digested with 0.1% trypsin(Invitrogen, USA) under gentle agitation for 30–50 min at 37(C. When tissue pieces became pale, they were washed three times, once with wash medium(DMEM/F12(3:1)(Invitrogen, USA) containing 1% penicillin/streptomycin(Cambrex, USA)) plus 10% fetal bovine serum and twice with Hank’s balanced salt solution. The epidermis was then removed from the dermis. Afterwards, dermis pieces were digested by collagenase type XI(Sigma-Aldrich, USA) for one hour at 37(C, mechanically dissociated with scissors and subsequently triturated repeatedly in wash medium with a 1000 μl pipette tip. The supernatant was collected and the trituration was repeated until tissue pieces became thin. After the dissociated cell suspension was filtered through a 40 μm cell strainer and centrifuged at 1200 rpm for 7 min, the pellet was suspended in proliferation medium(DMEM/F12(3:1) containing 0.1% penicillin/streptomycin, 40 ng/ml FGF2(Collaborative, USA), 20 ng/ml EGF(Collaborative, USA) and 2% B27 supplement(Invitrogen, USA)) to an optimum density of 10000–30000 cells/ml of medium. Finally, cells were cultured in proliferation medium at 37(C and used for experiments at the second generation. Cells were passaged every 3–4 days and used for experiments at the second generation. Primary fibroblasts(PFBs) were isolated from the dermis of neonatal BALB/c mice and cultured in DMEM plus 10% fetal bovine serum.

### Induction of Differentiation

For the induction of SKP differentiation into SFBs, SKP spheres were collected and suspended in DMEM plus 10% fetal bovine serum, and then plated onto a cell culture dish(Corning, USA) whose surface was coated with poly-L-lysine(Sigma-Aldrich, USA).

### Immunocytochemistry

Cells were fixed by 4% paraformaldehyde. The fixed cells were blocked with 3% BSA for 30 min and subsequently incubated with primary antibody overnight at 4℃. After washing with PBS 3 times, they were incubated with secondary antibody for 1 h at room temperature. Finally they were incubated with DAPI for 1 min. A parallel culture with secondary antibody only was employed as a negative control, a culture without any antibody was used as a blank control. Primary antibodies were monoclonal anti-fibronectin(Abcam, UK, 1:250), monoclonal anti-vimentin(Abcam, UK, 1:200), monoclonal anti-nestin(Abcam, UK, 1:500), monoclonal anti-SOX2(Abcam, UK, 1:200) and polyclonal anti-collagen 1(Abcam, UK, 1:500). Secondary antibodies were Alexa Fluor 488 goat-rabbit, Alexa Fluor 555 goat-rabbit and Alexa Fluor 488 goat-mouse(Invitrogen, USA, 1:500). The preceding protocol was performed in triplicate for each cell type described.

### RNA-Seq

**Library Preparation**. cDNA library preparation was performed at BGI-Shenzhen. The total RNA from SKPs and SFBs were firstly treated with DNase I to degrade contaminating DNA. mRNA was enriched by using oligo(dT) magnetic beads. The mRNA was broken into short fragments(about 200 bp) in fragmentation buffer. The RNA fragments were then ligated to adaptors and converted into cDNA, which was purified using magnetic beads. End reparation and 3’-end single nucleotide A(adenine) addition was then performed. Finally, sequencing adaptors were ligated to the fragments. The resulting fragments were enriched by PCR amplification. The library products were used for sequencing using IlluminaHiSeq^TM^ 2000. SKPs were considered as the control and SFBs were the treatment.

**Mapping Reads to the Reference Genome**. The original image data produced by the sequencer was transferred into sequences using base calling. These sequences were defined as “raw reads”. Prior to mapping these reads to the reference database, all sequences were filtered to remove adaptor sequences, N sequences(in which the percentage of unknown bases(N) was greater than 10%) and low-quality sequences(the percentages of low quality bases with a quality value ≤ 5 was greater than 50% in a read). The remaining reads were mapped to the mouse genome using SOAPaligner/SOAP2 [17]. No more than 2 mismatches were allowed in the alignment.

**Normalized Expression Levels of Genes and Screening of Differentially expressed genes(DEGs)**. The gene expression level was calculated by using RPKM [10] method(Reads Per Kb per Million reads). The used formula was as follows:

$$RPKM\left(A\right)=\frac{10^{6}C}{NL/10^{3}}$$

RPKM(A) is the expression level of gene A, C is the number of reads that uniquely aligned to gene A, N is the total number of reads that uniquely aligned to all genes, and L is the number of bases of gene A. The RPKM method is able to eliminate the influence of different gene length and sequencing discrepancies on the calculation of gene expression levels. Therefore, the RPKM values could be directly used for comparing the difference of gene expression among samples. The cutoff value for determining gene transcriptional activity was determined based on a 95% confidence interval for all RPKM values for each gene. A strict algorithm had been developed to identify DEGs between two samples based on “The significance of digital gene expression profiles” [18]. We used a P-value corresponding to a differential gene expression test at statistically significant levels [19]. ‘‘FDR(False Discovery Rate) ≤ 0.001 and the absolute value of log2Ratio ≥ 1” were used to identify DEGs as the threshold.

**Gene Ontology(GO) and KEGG Pathway Enrichment Analysis of DEGs**. GO enrichment analysis provides all GO terms that are significantly enriched in DEGs compared to the genome background, and filters the DEGs that correspond to biological functions. This method maps all DEGs to GO terms in the database(http://www.geneontology.org/), calculates gene numbers for every term, then uses hypergeometric test to find significantly enriched GO terms in DEGs compared to the genome background. The calculating formula is:

$$P=1-\sum_{i=0}^{m-1}\frac{\left(\begin{array}{l} M\\ i \end{array}\right)\left(\begin{array}{l} N-M\\ n-i \end{array}\right)}{\left(\begin{array}{l} N\\ n \end{array}\right)}$$

N is the number of all genes with GO annotation, n is the number of DEGs in N, M is the number of all genes that are annotated to the certain GO terms, m is the number of DEGs in M. The calculated p-value went through Bonferroni Correction, using a corrected p-value ≤ 0.05 as a threshold. GO annotation of DEGs was carried out using the Blast2GOprogram. After getting GO annotation for DEGs, we used WEGO software [20] to do GO functional classification for DEGs and to understand the distribution of gene functions of the species from the macro level. For KEGG annotation, which is the major public pathway-related database [21], the calculating formula is the same as that in GO analysis, where N is the number of all genes, n is the number of DEGs in N, M is the number of all genes annotated to specific pathways, and m was the number of DEGs in M.

**Software and Databases**. The software and databases used to analyze the RNA-Seq data are shown in Table 1.

**Table 1:** Software and Databases.

Analysis	Software/Algorithm (Version)	Database (Version)
**Statistics of alignment**	Soap(2.21)	
**Functional annotation**	BLAST(2.2.23); Blast2GO(2.2.5)	KEGG(updated monthly if possible); NR(updated monthly if possible); GO(updated monthly if possible)
**Quantification of gene expression**	RPKM algorithm	
**Screening of DEGs**	Poisson distribution model	
**Expression pattern analysis**	Cluster(3.0); Java Tree View(1.1.6r2)	
**Gene Ontology enrichment analysis**	Hypergeometric distribution model	GO(updated monthly if possible)
**Pathway enrichment analysis**	Hypergeometric distribution model	KEGG(updated monthly if possible)

### Real Time Quantitative Reverse Transcription PCR(qRT-PCR)

Main DEGs involved in cell differentiation and important signaling pathways were selected: *Bmp2*, *Kit*, *Id2*, *Bnc1*, *Wnt2b*, *Ptch2*, *Actg2*, *Wnt4*, *Myh11*, *Acta2*, *Smad6*, *Smad9*, *Myc*, *Megf6*, *Dner*, *Slitrk1*, *Lvl*, *Klk6*, *Agtr2*, *Tgfbr1*, *Smad3*, *Wnt11*, *Fzd10*, *Fzd4*, *Jun*, *Notch4*, *Notch1*, *Dlk1*, *Dtx4*. Total RNA was extracted using the RNeasy micro kit(Qiagen, Germany) and then was converted to cDNA using the SuperScript II Reverse Transcriptase kit(Invitrogen, USA) according to the manufacturer’s protocol. qRT-PCR was performed using a StepOnePlus Real-Time PCR System(Applied Biosystems) with Taqman primer/probe sets from Applied Biosystems. Three independent biological and two technical replicates were performed. mRNA expression levels were normalized by the internal β-actin control and then represented as the log2 ratio of the normalized values in fibroblasts to those in SKPs. Pearson correlation coefficient between qRT-PCR data and RNA-Seq data was calculated to validate RNA-Seq experiments. *T*-test was used to compare the gene expression between SFBs and PFBs and *P*< 0.01 was considered to be statistically significant.

## Results

### Differentiation of SKPs into Fibroblasts

**Fig 1:**
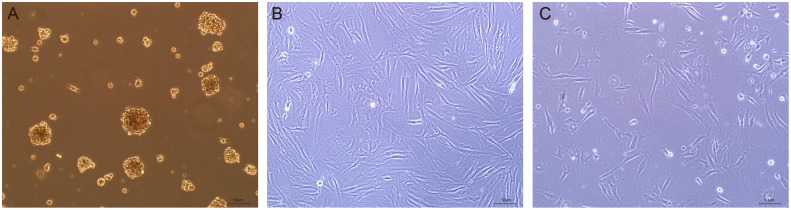
Morphology of SKPs and fibroblasts. (A) SKPs exhibited sphere-like structure in suspension culture.(B) PFBs were typically stellate or spindle shaped.(C) SFBs had the same morphology as PFBs.

**Fig 2:**
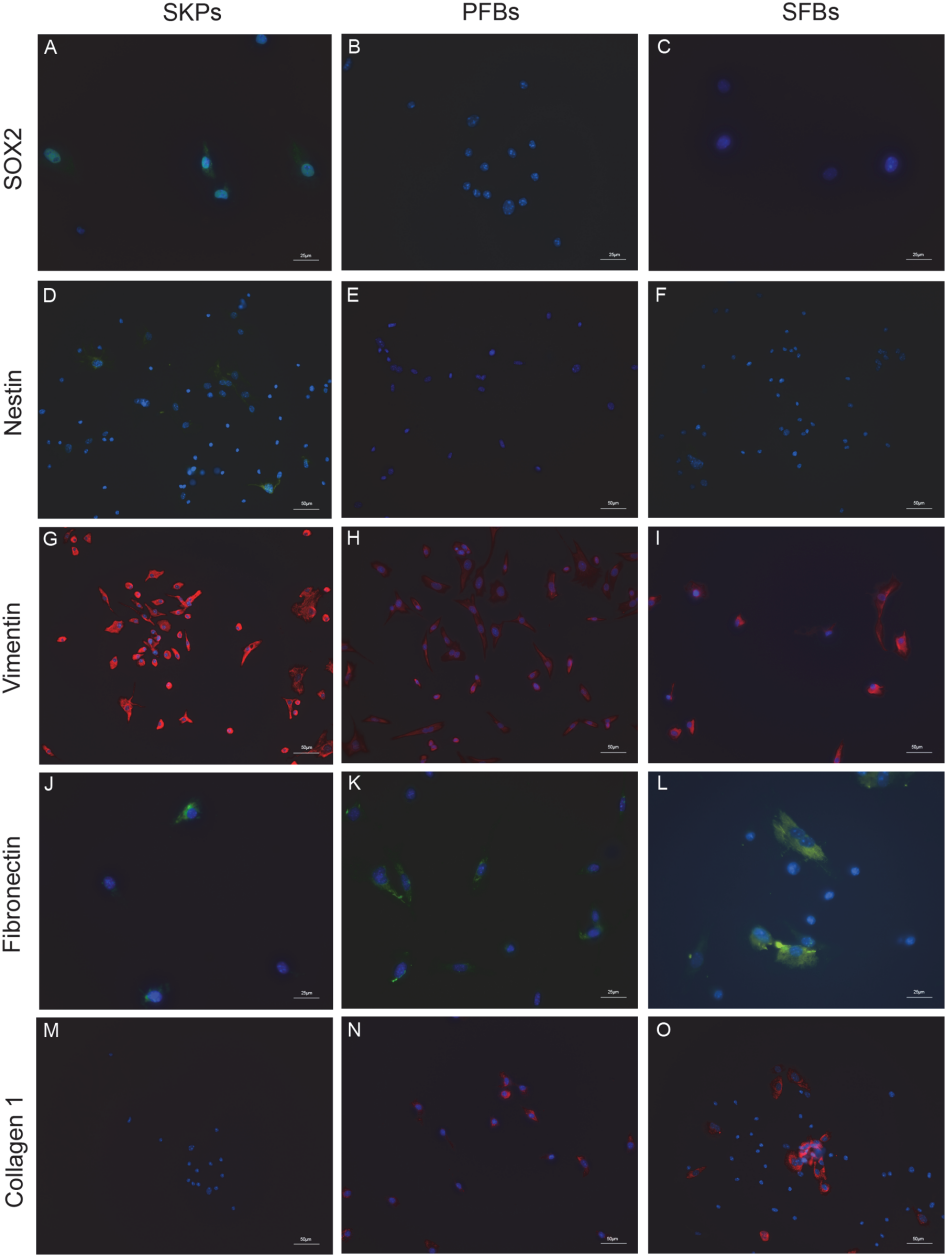
Characterization of SKPs and SFBs by immunocytochemistry. SKP spheres expressed SOX2(A), nestin(D), vimentin(G) and fibronectin(J), did not express collagen 1(M); SFBs expressed vimentin(I), fibronectin(L) and collagen 1(O), did not express SOX2(C) or nestin(F). The same results were observed with PFBs(B, E, H, K and N). Scale bars, 25 μm(A-C, J-L); 50 μm(D-I, M-O).

SKPs were successfully isolated from mouse dermal tissue and showed sphere-like structure in the suspension culture(Fig. 1A). SKPs attached to the bottom of poly-L-lysine treated dishes and exhibited fibroblast-like morphology(Fig. 1B and C) 3 days after serum induction. Consistent with previous studies [1, 8], SKP spheres expressed SOX2(Fig. 2A), nestin(Fig. 2D), vimentin(Fig. 2G) and fibronectin(Fig. 2J) when measured by immunocytochemistry. SFBs expressed fibroblast markers vimentin(Fig. 2I), fibronectin(Fig. 2L) and collagen 1(Fig. 2O), but did not express SOX2(Fig. 2C) or nestin(Fig. 2F). The same result was observed with PFBs(Fig. 2B, E, H, K and N). These results showed that SKPs could differentiate into fibroblasts, which is consistent with previous studies [6–8].

### Quality Assessment of Reads and Statistics of Alignment

**Fig 3:**
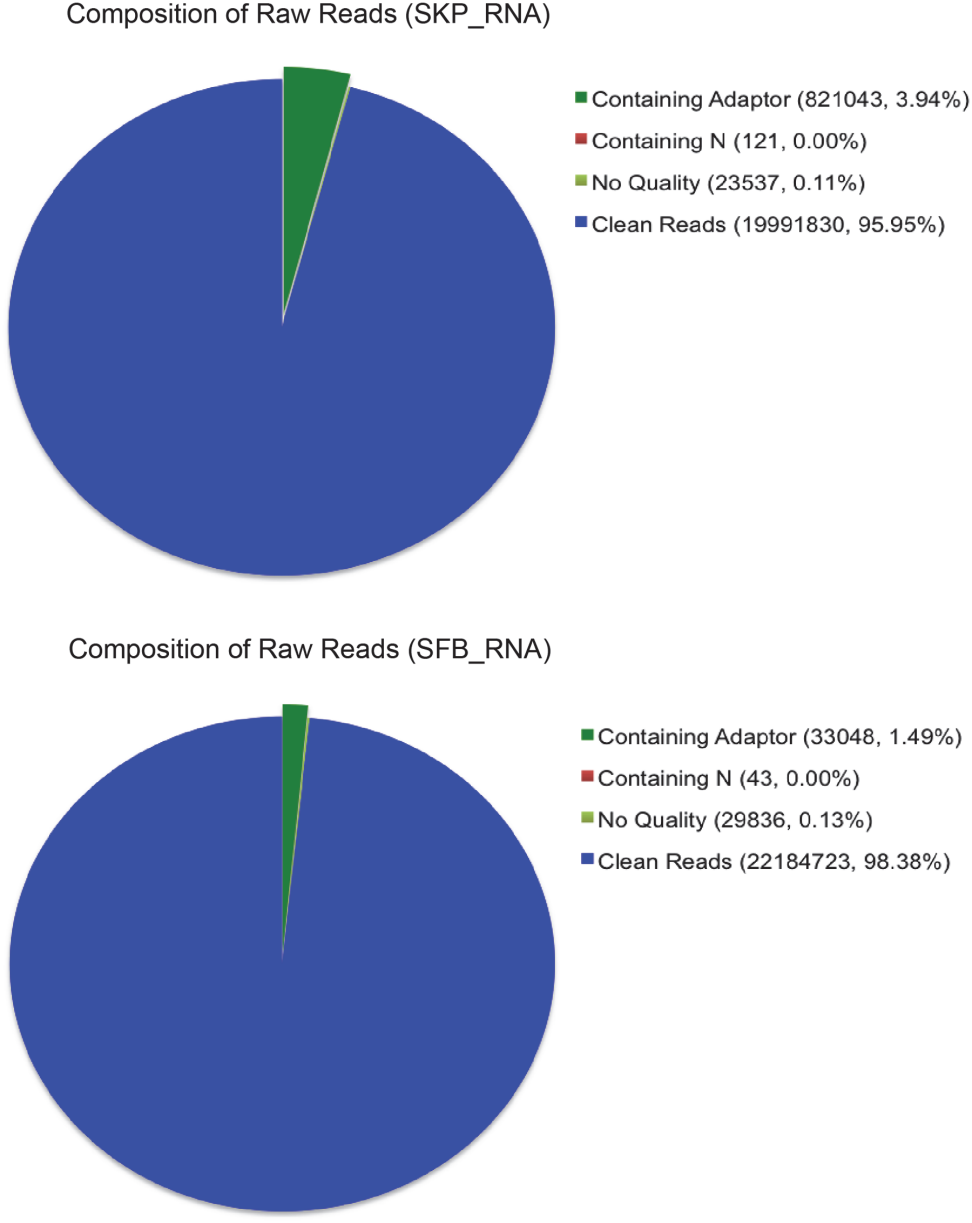
Classification of total raw reads. After filtering the only adaptor sequences, containing N sequences and low quality sequences, the RNA-Seq libraries of SKPs and SFBs generated over 19 million clean reads each, and the percentage of clean reads among raw tags in each library ranged from 95.95% to 98.38%.

To try to look into potential regulatory mechanisms that drive SKPs differentiate to SFBs, we performed RNA-Seq to measure RNA profiles in SKPs and SFBs. More than 20 million raw reads were generated from the SKPs or SFBs library. After filtering the only adaptor sequences, those containing N sequences and low quality sequences, the two RNA-Seq libraries still generated over 19 million clean reads from each library. The percentage of clean reads among raw tags in each library ranged from 95.95% to 98.38%(Fig. 3). Of the total reads, more than 84% matched to the mouse genome. The remaining sequences were unmatched(Table 2), because only reads aligning entirely inside exonic regions could be matched(reads from exon-exon junction regions could not be matched).

**Table 2:** Summary of mapping results(mapping to reference genes).

Sample ID	Total Reads	Total Base Pairs	Total Mapped Reads	Perfect Match	< = 2bp Mismatch	Unique Match	Multi-position Match	Total Unmapped Reads
**FB_RNA**	22184723(100.00%)	1087051427(100.00%)	18688411(84.24%)	15753372(71.01%)	2935039(13.23%)	14105047(63.58%)	4583364(20.66%)	3496312(15.76%)
**SKP_RNA**	19991830(100.00%)	979599327(100.00%)	17234956(86.21%%)	14226186(71.16%)	3008770(15.05%)	12832756(64.19%)	4402201(22.02%)	2756873(13.79%)

### Analysis of DEGs of SKPs and SFBs

**Fig 4:**
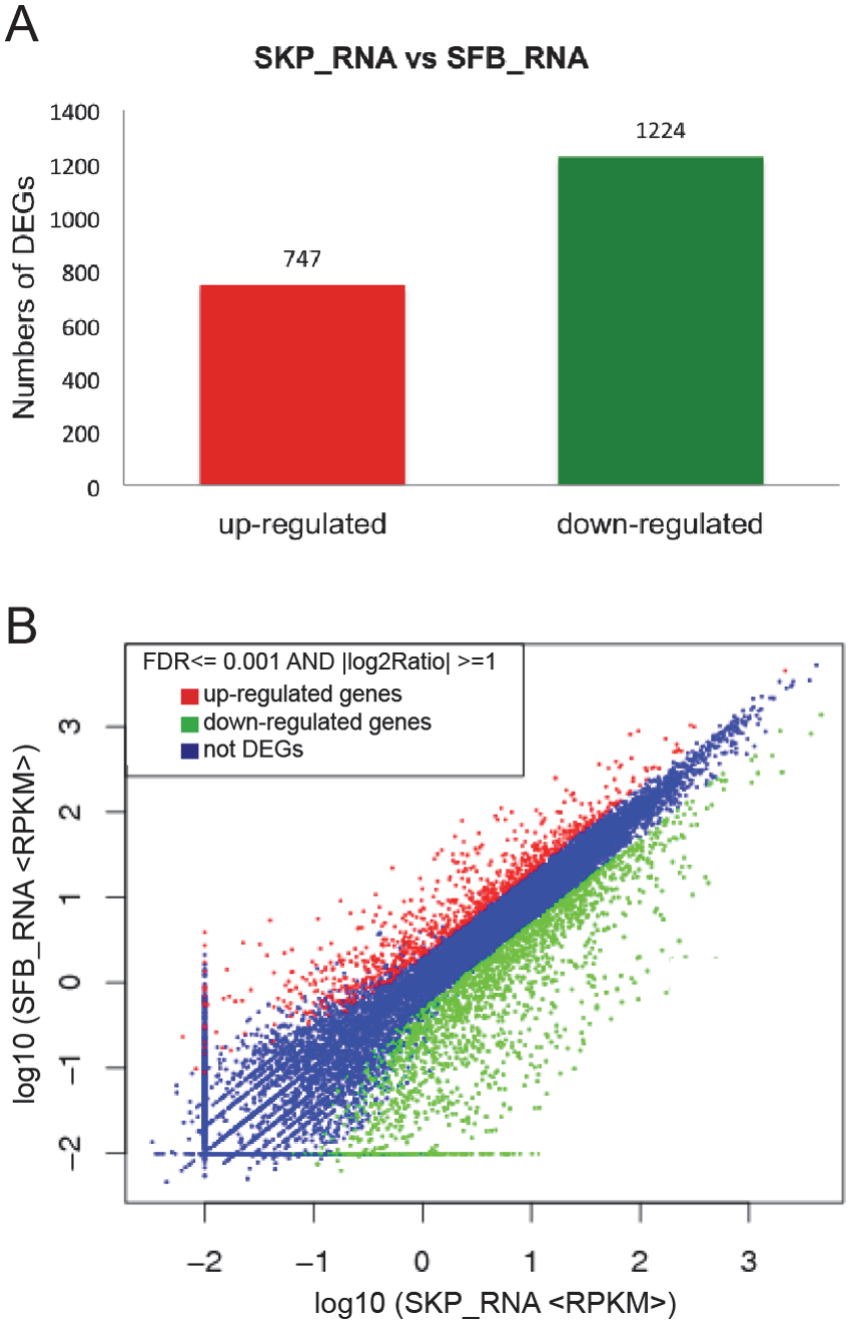
DEGs between SKPs and SFBs. (A) The numbers of DEGs.(B) Scattered plot of DEGs.

A total of 1971 genes were differentially expressed between SKPs and SFBs, with 747 genes up-regulated and 1224 down-regulated(Fig. 4).

### GO and KEGG pathway Enrichment Analysis of DEGs

**Fig 5:**
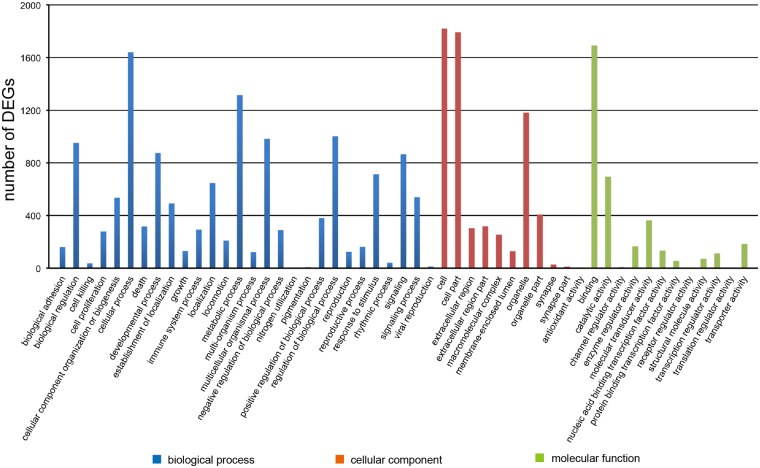
GO functional classification(WEGO) of DEGs. The results were summarized in three main domains: biological process, cellular component and molecular function. In the three main domains, 28, 10 and 13 functional groups were identified respectively.

GO is an international standardized gene functional classification system which offers a dynamic-updated controlled vocabulary and a strictly defined concept to comprehensively describe properties of genes and their products in any organism. GO covers three domains: cellular component, molecular function and biological process. The basic unit of GO is GO-term. Every GO-term belongs to a type of ontology. GO enrichment analysis provides all GO terms that are significantly enriched in DEGs compared to the genome background, and filters the DEGs that correspond to biological functions. In this study, 1971 DEGs could be categorized into 51 functional groups(Fig. 5). In the three main domains(biological process, cellular component and molecular function) of the GO classification, 28, 10 and 13 functional groups were identified, respectively. Among these groups, the terms cellular process, metabolic process and regulation of biological process in the biological process, the cell, cell part and organelle in the cellular component, the binding, catalytic activity and molecular transducer activity in the molecular function were dominant. Genes related to cell differentiation, cell proliferation, protein binding, transporter activity and membrane were also significantly enriched. The top five most up-regulated and down-regulated genes involved in these terms are listed in Table 3. Genes usually interact with each other to play roles in certain biological functions. Pathway-based analysis helps to further understand DEGs biological functions. KEGG pathway enrichment analysis identifies significantly enriched metabolic pathways or signal transduction pathways in DEGs compared with the whole genome background. 51 pathways were identified to be significantly enriched in DEGs between SKPs and SFBs(S1 Table). The most widely reported pathways related to stem cell pluripotency and differentiation, such as the TGF-β signaling pathway(S1 Fig.), Wnt signaling pathway(S2 Fig.) and Notch signaling pathway(S3 Fig.) were all included. The main DEGs involved in these signaling pathways are listed in Table 4.

**Table 3:** Most up-regulated and down-regulated genes involved in important terms about stem cells.

Term	Up-regulated genes	Down-regulated genes
**cell differentiation**	*Actg2*, *Wnt4*, *Myh11*, *Acta2*, *Tspan2*	*Slitrk1*, *Steap4*, *Ivl*, *Klk6*, *Agtr2*
**cell proliferation**	*Wisp2*, *Adra1d*, *Myocd*, *Thbs1*, *Tnfrsf11a*	*Camp*, *Thbs4*, *Msx1*, *Ace*, *IL15*
**protein binding**	*Wisp2*, *Myh11*, *Npas4*, *Wnt4*, *Cnn1*	*Lcn2*, *Thbs4*, *Stfa3*, *Agtr2*, *Sprr1b*
**transporter activity**	*Gabra4*, *Trpc6*, *Accn3*, *Slc13a5*, *Lrp2*	*Slc14a1*, *Atp6v0d2*, *Kcne1l*, *Clic5*, *Dmrt2*
**membrane**	*Slc13a5*, *Gal3st1*, *Wnt4*, *Lrp2*, *Adra1d*	*S100a8*, *Slitrk1*, *Atp6v0d2*, *Cd79b*, *Cidec*

**Table 4:** List of possible signaling pathways and major DEGs involved in these pathways.

**Signaling pathway**	Up-regulated DEGs	Down-regulated DEGs
**TGF-β signaling pathway**	*Gdf6*, *Tgfb3*, *Inhb*, *Bmpr2*, *Acvr2a*, *Smad6/9*, *Myc*, *Id3/4*	*Bmp2/4/6/7*, *Bmpr1*, *Tgfb1*, *Tgfbr1*, *Smad3*, *Cdkn2*, *Id2*
**Wnt signaling pathway**	*Wnt4*, *Wnt2b*, *Wnt9a*, *C-myc*	*Wnt7b*, *Wnt2*, *Wnt6*, *Wnt11*, *Fzd10*, *Fzd4*, *C-jun*, *Fra-1*, *Cyc-d*
**Notch signaling pathway**	*Megf6*, *Dner*	*Dlk1*, *Notch4*, *Sned1*, *Megf11*, *Notch3*, *Notch1*, *Notch2*, *Dtx1*, *Dtx4*, *Rbpj*

### qRT-PCR for Data Validation

**Fig 6:**
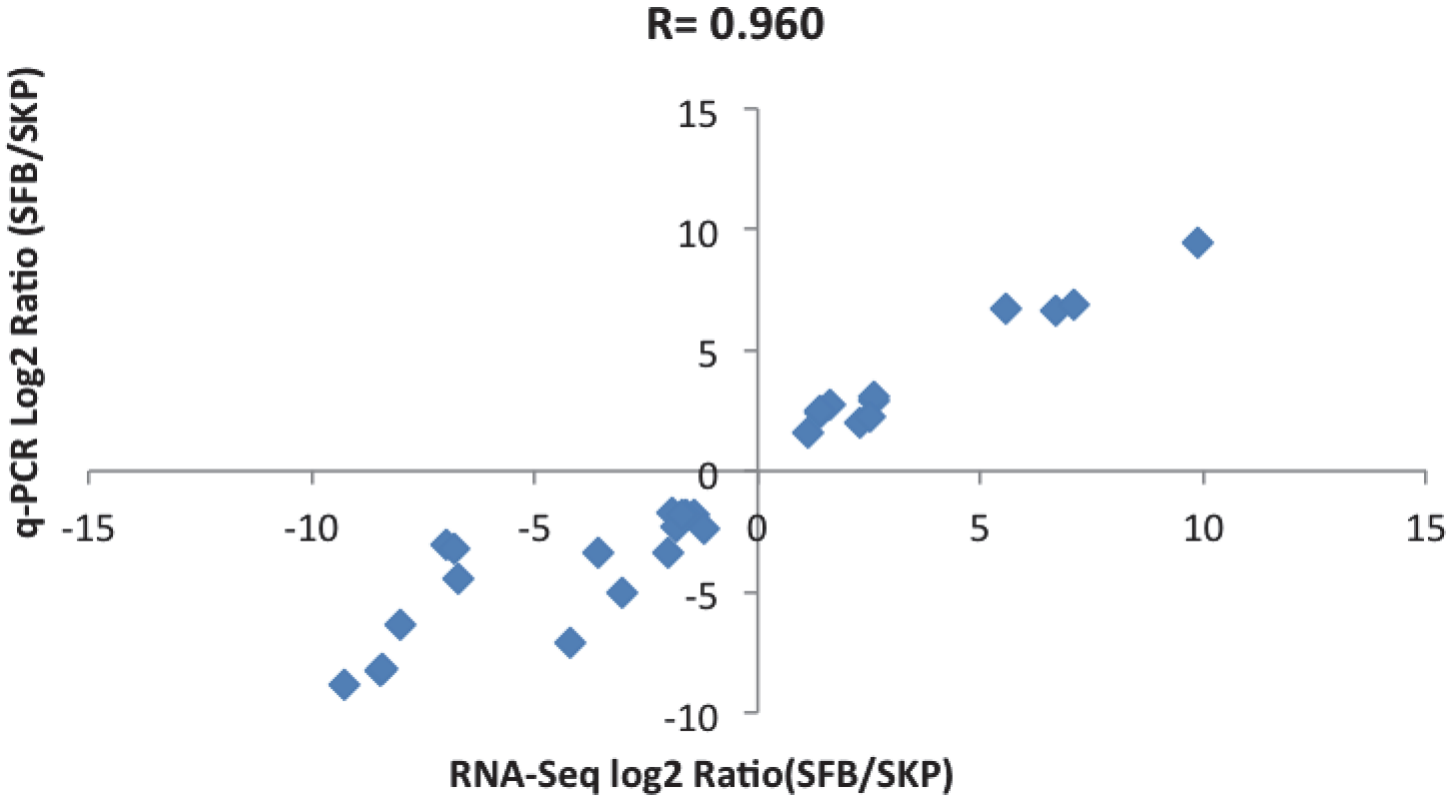
Correlation between RNA-Seq and qRT-PCR data of selected genes. Pearson correlation coefficient(r = 0.960) was used to determine the similarity in gene expression pattern between RNA-Seq and qRT-PCR.

**Fig 7:**
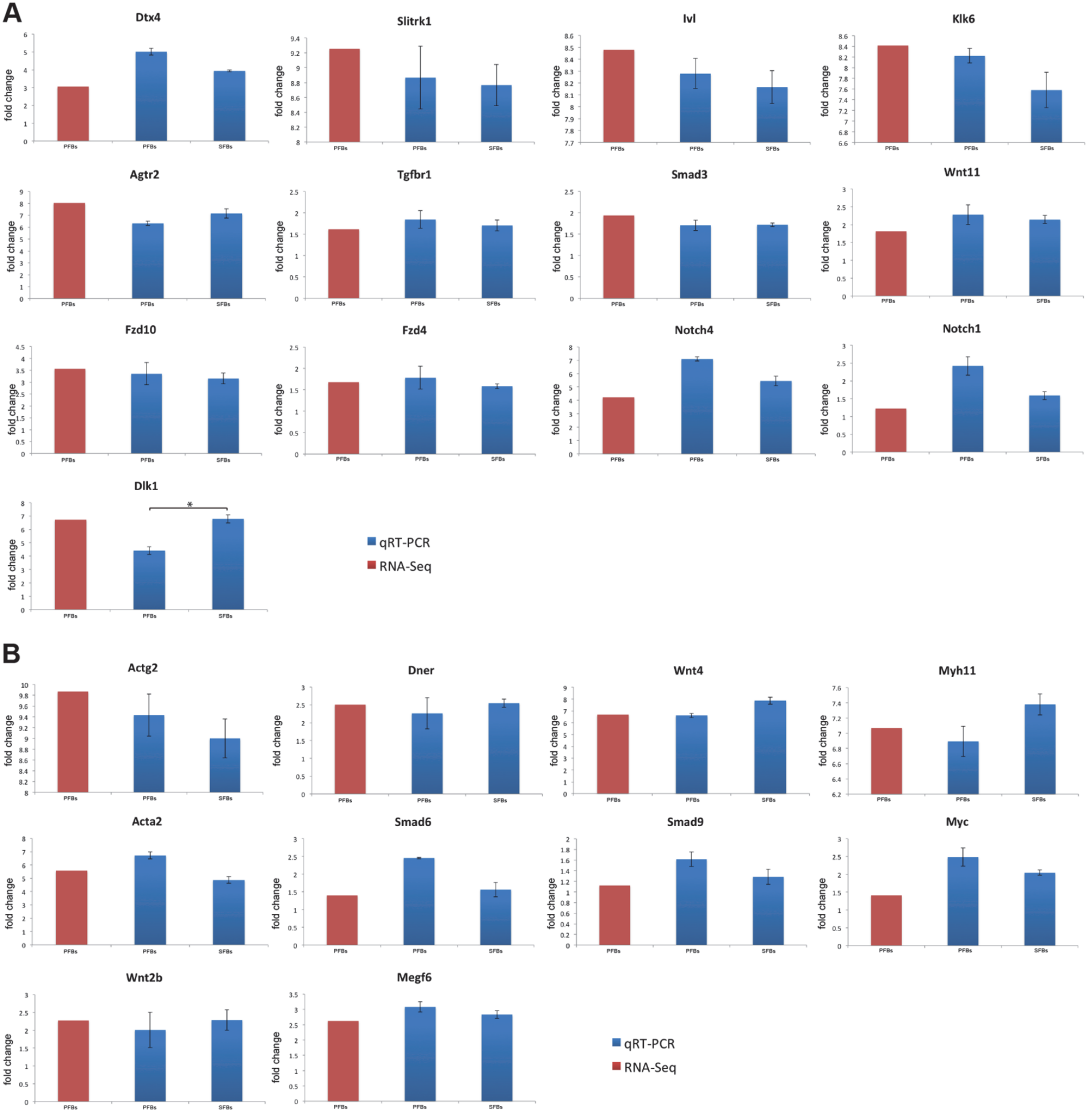
Validation of RNA-Seq results and comparison of gene expression between SFBs and PFBs by qRT-PCR. (A) Down-regulated genes.(B) Up-regulated genes. Fold changes shown are((SFBs or PFBs gene expression level)/(SKPs gene expression level)). Error bars represent SE; * represents statistically significant.

Main DEGs involved in cell differentiation and important signaling pathways were selected to verify the RNA-seq data by qRT-PCR. Pearson correlation coefficient between qRT-PCR data and RNA-Seq data was 0.960, which indicates that the RNA-Seq data is highly correlated with the qRT-PCR data(Fig. 6). PFBs were also tested by qRT-PCR. There was no significant difference in the expression of candidate genes between SFBs and PFBs except *Dlk1*(Fig. 7). Although the expression level of *Dlk1* between SFBs and PFBs was significantly different by qRT-PCR, it was down-regulated when compared with SKPs, which was consistent with the results from RNA-Seq. These results confirmed that RNA-Seq could provide reliable data for mRNA differential expression analysis.

## Disscussion

In this study we compared the transcriptional profiles of mouse SKPs and SFBs by RNA-Seq. Up to 1971 genes were found to be significantly differentially expressed, which was a much larger number than the finding by microarray [6]. The results suggest that RNA-Seq is a very sensitive tool to compare the gene expression between cells. GO analysis of the DEGs showed that these DEGs were significantly enriched in cell membrane and in the process of cell differentiation, cell proliferation, protein binding and transporter activity. Analysis of DEGs that are significantly enriched and involved in the above terms could help us identify the important candidate genes which might play important roles in the transition from SKPs to fibroblasts. Analysis of genes coding for cell membrane may also help us find the surface makers of SKPs and fibroblasts.

Among the listed up-regulated genes in table 3, the genes which encode WNT4 and WISP2 are both evidence of Wnt signaling activation [22–23]. The up-regulation of *Wnt4* and *Wisp2* indicates that the activation of the Wnt signaling pathway plays an important role in the transition from SKPs to fibroblasts. Thrombospondin-1(TSP-1), from the cell proliferation term, is an endogenous activator of TGF-β. TSP-1 has been reported to increase and decrease in parallel with that of TGF-β1 and collagen III [24]. TSP-1 also activates protein kinase B and decreases apoptotic signaling in suspended fibroblasts [25]. The up-regulation of the gene *Tsp1* indicates that it might help in inducing SKPs to differentiate into fibroblasts through the TGF-β signaling pathway by increasing the proliferation of SKPs.

Among the listed down-regulated genes, *Ivl* is related to epidermal cell differentiation and the protein encoded by it is synthesized in abundance during terminal differentiation of keratinocytes. ATP6V0D2 has been shown to be a regulator of osteoclast fusion and bone formation. The knock out of *Atp6v0d2* resulted in impaired osteoclast fusion and increased bone formation [26]. The down-regulation of these genes is related to keratinocytes and osteocytes, which indicates that the transition from SKPs to fibroblasts needs the inhibition of the genes that could lead to the differentiation to other cells. Many down-regulated genes have been reported to encode for functions in the neural system. The *Msx1* homeobox gene is expressed at diverse sites of epithelial-mesenchymal interaction during vertebrate embryogenesis, and has been implicated in signaling processes between tissue layers. It has a critical role in mediating epithelial-mesenchymal interactions during craniofacial bone and tooth development [27]. The down-regulation of *Msx1* may increase mesenchymal differentiation. *Slitrk1* encodes a transmembrane protein containing leucine-rich repeats that is produced predominantly in the nervous system. Previous work has shown that *Slitrk1*-deficient mice display elevated anxiety-like behavior and noradrenergic abnormalities [28]. KLK6 plays a functional role in oligodendrocyte development and the expression of myelin proteins [29]. AGTR2 regulates central nervous system functions, including behavior [30]. It also plays a role in the central nervous system and cardiovascular functions that are mediated by the renin-angiotensin system [31]. IL-15 is reported to be a key regulator of neurogenesis in the adult and is essential to understanding diseases with an inflammatory component [32]. The expression pattern of mouse *Kcne1l* in the developing embryo revealed a strong signal in ganglia, in the migrating neural crest cells of cranial nerves, in the somites, and in the myoepicardial layer of the heart. It was reported that KCNE1L could be involved in the development of some neurological signs observed in patients with AMME contiguous gene syndrome [33]. Inhibiting the expression of the down-regulated genes which encode the above proteins, which are related to the neural system, may induce SKPs to differentiate into mesodermal lineages rather than neural cells. Notch modulator THBS4 helps in rodent subventricular zone astrogenesis following injury [34]. The down-regulation of *Thbs4* suggests that the inhibition of the Notch signaling pathway may be involved in the process of transition from SKPs to fibroblasts.

Integration of extrinsic signals, epigenetic regulators, and intrinsic transcription factors underlies the capacity of stem cells to undergo differentiation. Differentiation is controlled by complex signaling networks. KEGG analysis of the DEGs showed that the major developmental signaling pathways including TGF-β signaling pathway, Wnt signaling pathway and Notch signaling pathway were all significantly enriched. These pathways were reported to be involved in the pluripotency and self-renewal of various stem cells.

The TGF-β signaling pathway is a series of molecular signals initiated by the binding of an extracellular ligand to a TGF-β receptor on the surface of a target cell, and ending with regulation of a downstream cellular process. The TGF-β superfamily comprises nearly 30 growth and differentiation factors that include TGF-βs, activins, inhibins, and bone morphogenetic proteins(BMPs). TGF-β inhibits proliferation of multipotent hematopoietic progenitors and promotes lineage commitment of neural precursors. BMPs block neural differentiation of mouse and human embryonic stem cells(ESCs) [35]. The TGF-β signaling pathway takes part in the differentiation process of various stem cells. Master differentiation genes in ESCs are secluded by repressive chromatin marks. TRIM33-Smad2/3 and Smad4-Smad2/3 complexes mediated TGF-β signals enable the transcriptional activation of ESCs [36]. Small interfering RNA experiments proved that TGF-β1 signaling through Smad2 and Smad3 plays an important role in the development of smooth muscle cells from totipotential ESCs [37]. TGF-β1 also suppresses ESC chondrogenic induction [38]. Canonical TGF-β signaling via Smad4 regulates the balance between proliferation and differentiation of neural stem cells in the midbrain [39]. The activation of TGF-β/BMP signaling pathway drives mesenchymal stem cells to differentiate into osteoblasts [40]. It also plays a crucial role in osteoblast differentiation of the adipose-derived stem cells. The members which are involved in the process include Smad 1, Smad5, Smad8, P38, ASK1, MKK3, MKK6, Runx2, collagen type 1, and osteopontin [41]. The TGF-β signaling pathway regulates the differentiation and proliferation of stem cells in different stages. In this study, The down-regulation of the gene *Smad3* led to up-regulation of transcription factors MYC and down-regulation of CDKN2 and ID2, which may be important in the differentiation from SKPs to fibroblasts. The Wnt signaling pathway was also reported to regulate the proliferation and differentiation of embryonic stem cells [42] and various kinds of adult stem cells [43–46]. In the Wnt signaling pathway, the up-regulated ligands WNT7B, WNT2, WNT6, WNT11 and the down-regulated WNT4 and WNT2B bound to their receptors, and led to the up-regulation of *C-myc* and down-regulation of *C-jun*, *Fra-1* and *Cyc-d*, which might also contribute to the differentiation of SKPs into fibroblasts. Notch signaling exhibits dynamic expression characteristics in bone marrow-derived mesenchymal stem cells during the process of differentiation into hepatocytes. It was found to be necessary to initiate differentiation into hepatocytes, but must be down-regulated for the differentiation to proceed continuously [47]. It has been shown that low adipogenic clones had significantly higher mRNA expression levels of *Notch2*, *Notch*3 and *Notch4*, *Jagged1*, as well as *Delta1*, compared with those of high adipogenic clones. This indicated that the activation of Notch signaling inhibited the adipogenic differentiation of adipose-derived mesenchymal stem cell clones [48]. Most of the DEGs involved in the Notch signaling pathway, including the genes encoding ligands, receptors and transcription factors, were down-regulated. These data suggest that the Notch signaling pathway plays an important role in keeping SKPs quiescent and the inhibition of this signaling pathway may activate SKPs to differentiate.

In conclusion, we compared the transcriptional profiles between SKPs and SFBs by RNA-Seq. GO analysis of the DEGs showed that the significantly up-regulated genes *Wnt4*, *Wisp2* and *Tsp-1* and the significantly down-regulated genes *Slitrk1*, *Klk6*, *Agtr2*, *Ivl*, *Msx1*, *IL15*, *Atp6v0d2*, *Kcne1l* and *Thbs4* might play important roles in the transition of SKPs to fibroblasts. KEGG analysis showed that DEGs were significantly enriched in the TGF-β signaling pathway, Wnt signaling pathway and Notch signaling pathway, which have been previously shown to regulate the differentiation and self-renewal of various stem cells. These identified DEGs and pathways could facilitate further investigations of the detailed molecular mechanisms, making it possible to take advantage of the potential therapeutic applications of SKPs in skin regeneration.

## Supporting Information

S1 Fig The detailed information of TGF-β signaling pathway. Up-regulated genes are marked with red borders and down-regulated genes with green borders.
(TIF)

S2 Fig The detailed information of Wnt signaling pathway. Up-regulated genes are marked with red borders and down-regulated genes with green borders.
(TIF)

S3 Fig The detailed information of Notch signaling pathway. Up-regulated genes are marked with red borders and down-regulated genes with green borders.
(TIF)

S1 Table KEGG pathway enrichment analysis of DEGs. (DOCX)
