# Supplementary figures and images for: Comparison of the Transcriptomes of Mouse Skin Derived Precursors (SKPs) and SKP-Derived Fibroblasts (SFBs) by RNA-Seq
Source: PLoS One. 2015 Feb 26;10(2):e0117739. doi: 10.1371/journal.pone.0117739 (PMC4342161; doi:10.1371/journal.pone.0117739)

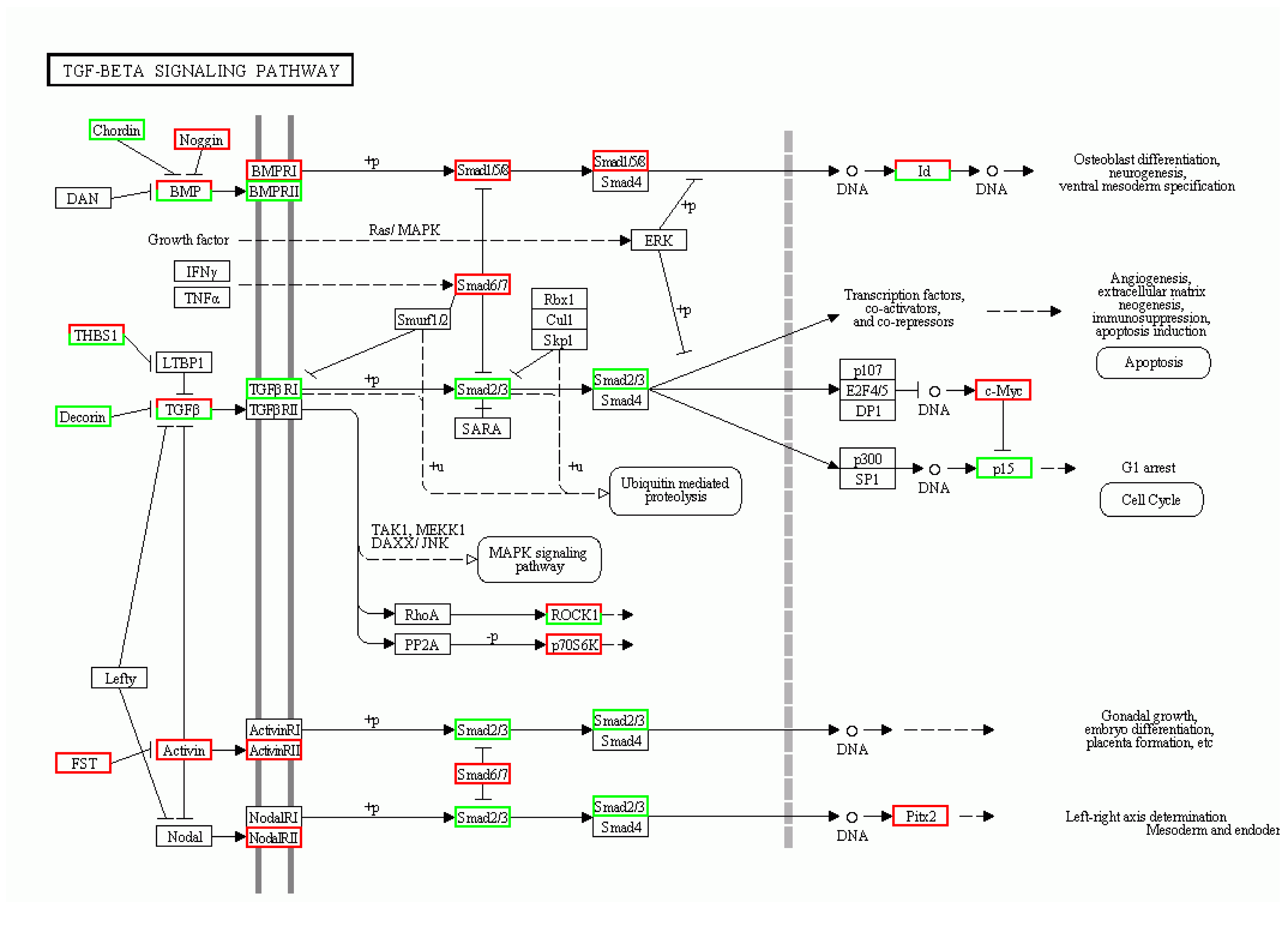

Supplement: S1 Fig — Up-regulated genes are marked with red borders and down-regulated genes with green borders. (TIF) [file pone.0117739.s001.tif]

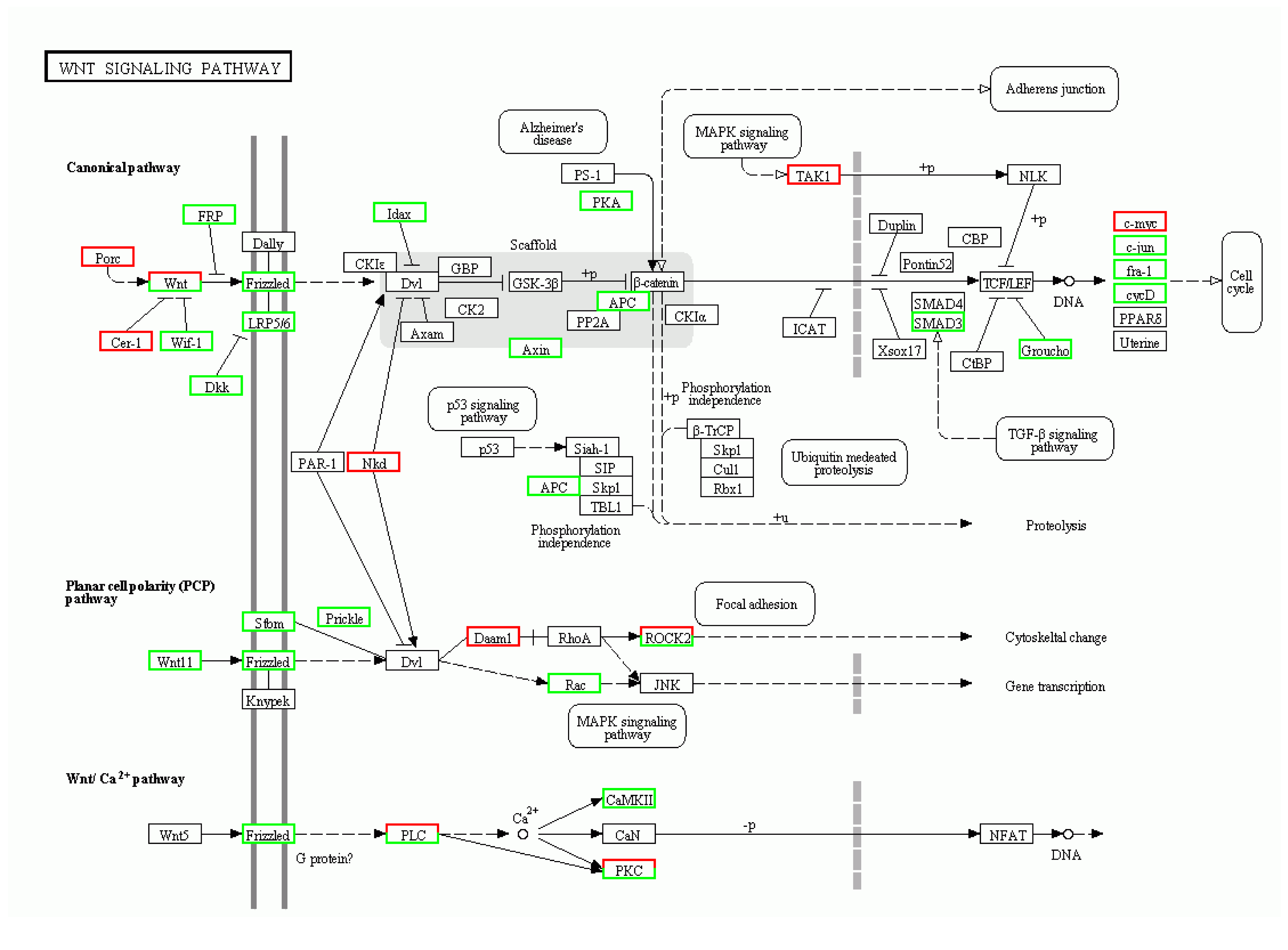

Supplement: S2 Fig — Up-regulated genes are marked with red borders and down-regulated genes with green borders. (TIF) [file pone.0117739.s002.tif]

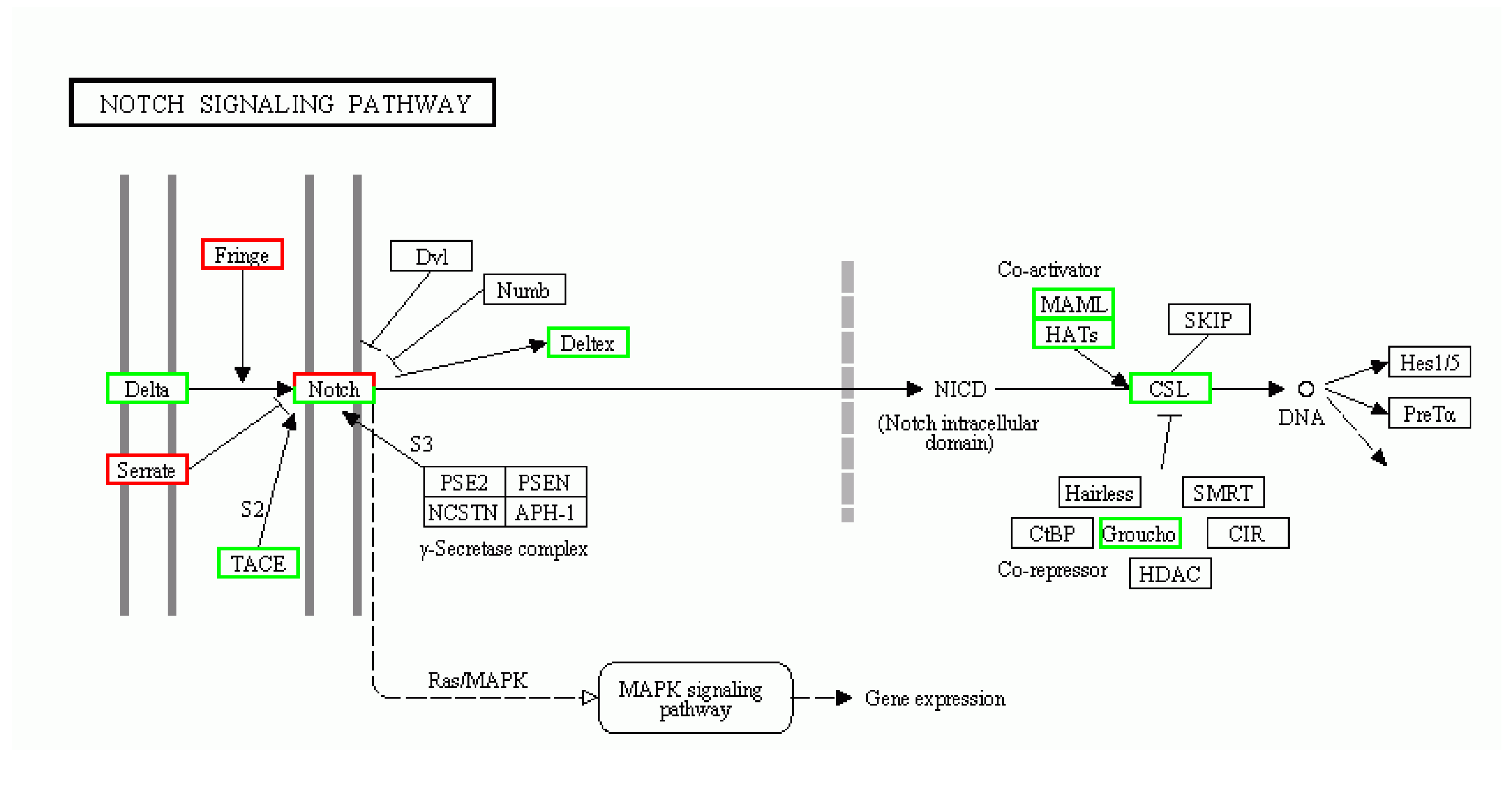

Supplement: S3 Fig — Up-regulated genes are marked with red borders and down-regulated genes with green borders. (TIF) [file pone.0117739.s003.tif]
