# Supplementary material for: Comparison of the Transcriptomes of Mouse Skin Derived Precursors (SKPs) and SKP-Derived Fibroblasts (SFBs) by RNA-Seq
Source: PLoS One. 2015 Feb 26;10(2):e0117739. doi: 10.1371/journal.pone.0117739 (PMC4342161; doi:10.1371/journal.pone.0117739)
Supplement: S1 Table — (DOCX) [file pone.0117739.s004.docx]

S1 Table. KEGG pathway enrichment analysis of DEGs

| # | Pathway | DEGs with pathway annotation (2196) | All genes with pathway annotation (16839) | P value | Q value |
| --- | --- | --- | --- | --- | --- |
| 1 | [ECM-receptor interaction](file:///F:\doctor\RNA-SEQ%20Report\upload\GeneDiffExp\Pathway\SKP_RNA-VS-FB_RNA.htm#gene1) | 69 (3.14%) | 200 (1.19%) | 1.283391e-15 | 3.117396e-13 |
| 2 | [Focal adhesion](file:///F:\\doctor\\RNA-SEQ%20Report\\upload\\GeneDiffExp\\Pathway\\SKP_RNA-VS-FB_RNA.htm#gene2) | 106 (4.83%) | 409 (2.43%) | 2.637115e-12 | 3.191220e-10 |
| 3 | [Axon guidance](file:///F:\\doctor\\RNA-SEQ%20Report\\upload\\GeneDiffExp\\Pathway\\SKP_RNA-VS-FB_RNA.htm#gene3) | 80 (3.64%) | 295 (1.75%) | 8.978279e-12 | 7.272157e-10 |
| 4 | [TGF-beta signaling pathway](file:///F:\\doctor\\RNA-SEQ%20Report\\upload\\GeneDiffExp\\Pathway\\SKP_RNA-VS-FB_RNA.htm#gene4) | 46 (2.09%) | 142 (0.84%) | 5.267290e-10 | 3.167641e-08 |
| 5 | [Amoebiasis](file:///F:\\doctor\\RNA-SEQ%20Report\\upload\\GeneDiffExp\\Pathway\\SKP_RNA-VS-FB_RNA.htm#gene5) | 59 (2.69%) | 208 (1.24%) | 2.274293e-08 | 1.105432e-06 |
| 6 | [Cytokine-cytokine receptor interaction](file:///F:\\doctor\\RNA-SEQ%20Report\\upload\\GeneDiffExp\\Pathway\\SKP_RNA-VS-FB_RNA.htm#gene6) | 81 (3.69%) | 348 (2.07%) | 9.597264e-08 | 3.886892e-06 |
| 7 | [Protein digestion and absorption](file:///F:\doctor\RNA-SEQ%20Report\upload\GeneDiffExp\Pathway\SKP_RNA-VS-FB_RNA.htm#gene7) | 43 (1.96%) | 153 (0.91%) | 5.530518e-07 | 1.919880e-05 |
| 8 | [Pathways in cancer](file:///F:\\doctor\\RNA-SEQ%20Report\\upload\\GeneDiffExp\\Pathway\\SKP_RNA-VS-FB_RNA.htm#gene8) | 100 (4.55%) | 534 (3.17%) | 1.592714e-06 | 4.837869e-05 |
| 9 | [Salivary secretion](file:///F:\\doctor\\RNA-SEQ%20Report\\upload\\GeneDiffExp\\Pathway\\SKP_RNA-VS-FB_RNA.htm#gene9) | 40 (1.82%) | 147 (0.87%) | 3.292051e-06 | 8.885568e-05 |
| 10 | [Dilated cardiomyopathy](file:///F:\doctor\RNA-SEQ%20Report\upload\GeneDiffExp\Pathway\SKP_RNA-VS-FB_RNA.htm#gene10) | 64 (2.91%) | 283 (1.68%) | 5.726986e-06 | 1.318644e-04 |
| 11 | [Arrhythmogenic right ventricular cardiomyopathy (ARVC)](file:///F:\doctor\RNA-SEQ%20Report\upload\GeneDiffExp\Pathway\SKP_RNA-VS-FB_RNA.htm#gene11) | 37 (1.68%) | 136 (0.81%) | 7.612465e-06 | 1.679121e-04 |
| 12 | [Complement and coagulation cascades](file:///F:\doctor\RNA-SEQ%20Report\upload\GeneDiffExp\Pathway\SKP_RNA-VS-FB_RNA.htm#gene12) | 45 (2.05%) | 180 (1.07%) | 9.637251e-06 | 1.924717e-04 |
| 13 | [Staphylococcus aureus infection](file:///F:\doctor\RNA-SEQ%20Report\upload\GeneDiffExp\Pathway\SKP_RNA-VS-FB_RNA.htm#gene13) | 32 (1.46%) | 112 (0.67%) | 1.029684e-05 | 1.926117e-04 |
| 14 | [MAPK signaling pathway](file:///F:\doctor\RNA-SEQ%20Report\upload\GeneDiffExp\Pathway\SKP_RNA-VS-FB_RNA.htm#gene14) | 89 (4.05%) | 403 (2.39%) | 1.250723e-05 | 2.123316e-04 |
| 15 | [Malaria](file:///F:\doctor\RNA-SEQ%20Report\upload\GeneDiffExp\Pathway\SKP_RNA-VS-FB_RNA.htm#gene15) | 25 (1.14%) | 79 (0.47%) | 1.338631e-05 | 2.129316e-04 |
| 16 | [PPAR signaling pathway](file:///F:\doctor\RNA-SEQ%20Report\upload\GeneDiffExp\Pathway\SKP_RNA-VS-FB_RNA.htm#gene16) | 39 (1.78%) | 150 (0.89%) | 1.400965e-05 | 2.127716e-04 |
| 17 | [Bladder cancer](file:///F:\doctor\RNA-SEQ%20Report\upload\GeneDiffExp\Pathway\SKP_RNA-VS-FB_RNA.htm#gene17) | 21 (0.96%) | 63 (0.37%) | 2.748383e-05 | 3.922382e-04 |
| 18 | [Regulation of actin cytoskeleton](file:///F:\doctor\RNA-SEQ%20Report\upload\GeneDiffExp\Pathway\SKP_RNA-VS-FB_RNA.htm#gene18) | 98 (4.46%) | 506 (3%) | 3.123338e-05 | 3.989328e-04 |
| 19 | [Hypertrophic cardiomyopathy (HCM)](file:///F:\doctor\RNA-SEQ%20Report\upload\GeneDiffExp\Pathway\SKP_RNA-VS-FB_RNA.htm#gene19) | 61 (2.78%) | 280 (1.66%) | 3.123841e-05 | 3.995228e-04 |
| 20 | [Renin-angiotensin system](file:///F:\doctor\RNA-SEQ%20Report\upload\GeneDiffExp\Pathway\SKP_RNA-VS-FB_RNA.htm#gene20) | 12 (0.55%) | 26 (0.15%) | 3.852588e-05 | 4.680894e-04 |
| 21 | [Vascular smooth muscle contraction](file:///F:\doctor\RNA-SEQ%20Report\upload\GeneDiffExp\Pathway\SKP_RNA-VS-FB_RNA.htm#gene21) | 70 (3.19%) | 339 (2.01%) | 5.344531e-05 | 6.024057e-04 |
| 22 | [Calcium signaling pathway](file:///F:\\doctor\\RNA-SEQ%20Report\\upload\\GeneDiffExp\\Pathway\\SKP_RNA-VS-FB_RNA.htm#gene22) | 53 (2.41%) | 238 (1.41%) | 5.453384e-05 | 6.024156e-04 |
| 23 | [Cell adhesion molecules (CAMs)](file:///F:\doctor\RNA-SEQ%20Report\upload\GeneDiffExp\Pathway\SKP_RNA-VS-FB_RNA.htm#gene23) | 51 (2.32%) | 223 (1.32%) | 7.282029e-05 | 7.693622e-04 |
| 24 | [Hematopoietic cell lineage](file:///F:\doctor\RNA-SEQ%20Report\upload\GeneDiffExp\Pathway\SKP_RNA-VS-FB_RNA.htm#gene24) | 34 (1.55%) | 137 (0.81%) | 0.0001314311 | 1.330740e-03 |
| 25 | [Rheumatoid arthritis](file:///F:\doctor\RNA-SEQ%20Report\upload\GeneDiffExp\Pathway\SKP_RNA-VS-FB_RNA.htm#gene25) | 29 (1.32%) | 111 (0.66%) | 0.0001494109 | 1.497942e-03 |
| 26 | [p53 signaling pathway](file:///F:\doctor\RNA-SEQ%20Report\upload\GeneDiffExp\Pathway\SKP_RNA-VS-FB_RNA.htm#gene26) | 32 (1.46%) | 129 (0.77%) | 0.0002060819 | 1.926127e-03 |
| 27 | [Phagosome](file:///F:\doctor\RNA-SEQ%20Report\upload\GeneDiffExp\Pathway\SKP_RNA-VS-FB_RNA.htm#gene27) | 67 (3.05%) | 344 (2.04%) | 0.0002620851 | 2.356933e-03 |
| 28 | [Melanogenesis](file:///F:\doctor\RNA-SEQ%20Report\upload\GeneDiffExp\Pathway\SKP_RNA-VS-FB_RNA.htm#gene28) | 31 (1.41%) | 131 (0.78%) | 0.0006171029 | 5.353696e-03 |
| 29 | [Chemokine signaling pathway](file:///F:\doctor\RNA-SEQ%20Report\upload\GeneDiffExp\Pathway\SKP_RNA-VS-FB_RNA.htm#gene29) | 56 (2.55%) | 280 (1.66%) | 0.0006320614 | 5.4633285e-03 |
| 30 | [Steroid biosynthesis](file:///F:\doctor\RNA-SEQ%20Report\upload\GeneDiffExp\Pathway\SKP_RNA-VS-FB_RNA.htm#gene30) | 10 (0.46%) | 25 (0.15%) | 0.000750096 | 5.711398e-03 |
| 31 | [Nicotinate and nicotinamide metabolism](file:///F:\doctor\RNA-SEQ%20Report\upload\GeneDiffExp\Pathway\SKP_RNA-VS-FB_RNA.htm#gene31) | 14 (0.64%) | 43 (0.26%) | 0.0007684383 | 6.123049e-03 |
| 32 | [Neuroactive ligand-receptor interaction](file:///F:\doctor\RNA-SEQ%20Report\upload\GeneDiffExp\Pathway\SKP_RNA-VS-FB_RNA.htm#gene32) | 68 (3.1%) | 359 (2.13%) | 0.000899355 | 6.755811e-03 |
| 33 | [Glutamatergic synapse](file:///F:\doctor\RNA-SEQ%20Report\upload\GeneDiffExp\Pathway\SKP_RNA-VS-FB_RNA.htm#gene33) | 36 (1.64%) | 163 (0.97%) | 0.0009281797 | 6.903351e-03 |
| 34 | [Gap junction](file:///F:\doctor\RNA-SEQ%20Report\upload\GeneDiffExp\Pathway\SKP_RNA-VS-FB_RNA.htm#gene34) | 28 (1.28%) | 118 (0.7%) | 0.001054435 | 7.543286e-03 |
| 35 | [Gastric acid secretion](file:///F:\doctor\RNA-SEQ%20Report\upload\GeneDiffExp\Pathway\SKP_RNA-VS-FB_RNA.htm#gene35) | 34 (1.55%) | 153 (0.91%) | 0.001132151 | 7.929320e-03 |
| 36 | [Prion diseases](file:///F:\doctor\RNA-SEQ%20Report\upload\GeneDiffExp\Pathway\SKP_RNA-VS-FB_RNA.htm#gene36) | 23 (1.05%) | 92 (0.55%) | 0.001361549 | 9.190456e-03 |
| 37 | [Chagas disease (American trypanosomiasis)](file:///F:\doctor\RNA-SEQ%20Report\upload\GeneDiffExp\Pathway\SKP_RNA-VS-FB_RNA.htm#gene37) | 31 (1.41%) | 138 (0.82%) | 0.001535462 | 1.008425e-02 |
| 38 | [Leukocyte transendothelialmigration](file:///F:\doctor\RNA-SEQ%20Report\upload\GeneDiffExp\Pathway\SKP_RNA-VS-FB_RNA.htm#gene38) | 48 (2.19%) | 243 (1.44%) | 0.001987778 | 1.271132e-02 |
| 39 | [Endocrine and other factor-regulated calcium reabsorption](file:///F:\doctor\RNA-SEQ%20Report\upload\GeneDiffExp\Pathway\SKP_RNA-VS-FB_RNA.htm#gene39) | 21 (0.96%) | 84 (0.5%) | 0.002156068 | 1.343396e-02 |
| 40 | [Wnt signaling pathway](file:///F:\doctor\RNA-SEQ%20Report\upload\GeneDiffExp\Pathway\SKP_RNA-VS-FB_RNA.htm#gene40) | 47 (2.14%) | 235 (1.4%) | 0.002939101 | 1.776998e-02 |
| 41 | [Proximal tubule bicarbonate reclamation](file:///F:\doctor\RNA-SEQ%20Report\upload\GeneDiffExp\Pathway\SKP_RNA-VS-FB_RNA.htm#gene41) | 13 (0.59%) | 44 (0.26%) | 0.003119167 | 1.848677e-02 |
| 42 | [Adherens junction](file:///F:\doctor\RNA-SEQ%20Report\upload\GeneDiffExp\Pathway\SKP_RNA-VS-FB_RNA.htm#gene42) | 32 (1.46%) | 152 (0.9%) | 0.003857296 | 2.231721e-02 |
| 43 | [Notch signaling pathway](file:///F:\doctor\RNA-SEQ%20Report\upload\GeneDiffExp\Pathway\SKP_RNA-VS-FB_RNA.htm#gene43) | 19 (0.87%) | 77 (0.46%) | 0.004022837 | 2.262068e-02 |
| 44 | [Terpenoid backbone biosynthesis](file:///F:\doctor\RNA-SEQ%20Report\upload\GeneDiffExp\Pathway\SKP_RNA-VS-FB_RNA.htm#gene44) | 12 (0.55%) | 26 (0.15%) | 0.004822072 | 2.298582e-02 |
| 45 | [NF-kappa B signaling pathway](file:///F:\doctor\RNA-SEQ%20Report\upload\GeneDiffExp\Pathway\SKP_RNA-VS-FB_RNA.htm#gene45) | 33 (1.50%) | 172 (1.02%) | 0.004624531 | 2.497247e-02 |
| 46 | [Morphine addiction](file:///F:\doctor\RNA-SEQ%20Report\upload\GeneDiffExp\Pathway\SKP_RNA-VS-FB_RNA.htm#gene46) | 25 (1.14%) | 113 (0.67%) | 0.005129595 | 2.709764e-02 |
| 47 | [HTLV-I infection](file:///F:\doctor\RNA-SEQ%20Report\upload\GeneDiffExp\Pathway\SKP_RNA-VS-FB_RNA.htm#gene48) | 71 (3.23%) | 389 (2.31%) | 0.006773846 | 3.429863e-02 |
| 48 | [Hedgehog signaling pathway](file:///F:\\doctor\\RNA-SEQ%20Report\\upload\\GeneDiffExp\\Pathway\\SKP_RNA-VS-FB_RNA.htm#gene49) | 17 (0.77%) | 70 (0.42%) | 0.00733514 | 3.633122e-02 |
| 49 | [Transcriptional misregulation in cancer](file:///F:\doctor\RNA-SEQ%20Report\upload\GeneDiffExp\Pathway\SKP_RNA-VS-FB_RNA.htm#gene51) | 65 (2.96%) | 371 (2.2%) | 0.00710328 | 3.612311e-02 |
| 50 | [Pancreatic secretion](file:///F:\doctor\RNA-SEQ%20Report\upload\GeneDiffExp\Pathway\SKP_RNA-VS-FB_RNA.htm#gene52) | 36 (1.64%) | 172 (1.02%) | 0.00816745 | 3.831773e-02 |
| 51 | [Leishmaniasis](file:///F:\doctor\RNA-SEQ%20Report\upload\GeneDiffExp\Pathway\SKP_RNA-VS-FB_RNA.htm#gene53) | 23 (1.04%) | 100 (0.59%) | 0.008931715 | 4.072183e-02 |
